# Assessment of exonic single nucleotide polymorphisms in the adenosine A_2A_ receptor gene to high myopia susceptibility in Chinese subjects

**Published:** 2011-02-16

**Authors:** Xiaoyan Chen, Anquan Xue, Wei Chen, Yang Ding, Dongsheng Yan, Jiqing Peng, Changqing Zeng, Jia Qu, Xiangtian Zhou

**Affiliations:** 1 School of Optometry and Ophthalmology and Eye Hospital, Wenzhou Medical College, Wenzhou, Zhejiang, China; 2 State Key Laboratory Cultivation Base and Key Laboratory of Vision Science, Ministry of Health P.R.China and Zhejiang Provincial Key Laboratory of Ophthalmology and Optometry, Wenzhou, Zhejiang, China; 3 Beijing Institute of Genomics, Chinese Academy of Sciences, Beijing, China; 4 Graduate School of the Chinese Academy of Sciences, Beijing, China

## Abstract

**Purpose:**

The adenosine A_2A_ receptor (A_2A_R) modulates collagen synthesis and extracellular matrix production in ocular tissues that contribute to eye growth and the development of myopia. We aimed to determine if single nucleotide polymorphisms (SNPs) in *A_2A_R* exons associates with high myopia found in Chinese subjects.

**Methods:**

DNA samples were prepared from venous lymphocytes of 175 Chinese subjects with high myopia of less than –8.00 diopters (D) correction and 101 ethnically similar controls with between –1.00 D and +1.00 D correction. The coding region sequences of *A_2A_R* were amplified by PCR and analyzed by Sanger sequencing. The detected variations were confirmed by reverse sequencing. Allelic frequencies of all detected common SNPs were assessed for Hardy–Weinberg equilibrium.

**Results:**

Five variations in *A_2A_R* exons, 5675 A>G, 5765 C>T, 13325 G>A, 13448 C>T, and 14000 T>A, were detected in controls at a low frequency (<1%). However, one SNP, 13772 T>C (rs5751876), showed its polymorphism in 53.3% of the total study population. The rs5751876 is a synonymous substitution located in a tyrosine codon of exon 2. Despite no significant difference in genotype distribution between cases and controls, the frequency of heterozygotes with the rs5751876 genotype was significantly lower in subjects with high myopia.

**Conclusions:**

The reduced frequency of the heterozygote rs5751876 genotype in subjects suggests a possible association of *A_2A_R* with high myopia in a Chinese population.

## Introduction

Human high myopia is an extreme form of myopia with an excessive increase in the ocular axial length and with pathological structural changes in the sclera. These changes include thinning of sclera due to reduced collagen production and tissue loss in the posterior segment [1-3]. The exact mechanism that causes such abnormal eye development is unknown. It is now widely recognized that in addition to a series of biologic factors and neurotransmitter pathways [4-11], genetic factors affecting transcription and other regulatory activities contribute to the complex process of ocular development and the occurrence of high myopia [12-15].

The role of adenosine in vision development and myopia formation has recently been reported. In the tiger salamander, the adenosine A_2A_ receptor (A_2A_R) regulates vision formation by mediating inhibition of rod opsin mRNA expression [16]. In form deprivation myopic guinea pigs, adenosine A_2A_R is increased in the retina, choroid, and sclera [17]. In addition, treatment with the adenosine receptor antagonist 7-methylxanthine decreases myopia progression and eye elongation in myopic children and in form deprivation myopic knockout guinea pigs [18,19]. In previous findings we detected a greater myopic shift with longer axial length, increased vitreous chamber depth, and altered scleral collagen fiber structure in *A_2A_R* knockout mice compared to wild-type littermates [20]. We also found that the A_2A_R agonist CGS21680 increases the expression of collagen I, III, and V mRNAs in cultured human scleral fibroblasts and promotes production of soluble collagen in a concentration-dependent manner [20].

Adenosine is the product of ATP metabolism present in all cells and body fluids, where it also acts as an important neurotransmitter and an effective vasodilator [21]. It elicits biologic responses by binding to adenosine receptors, including A_1_, A_2A_, A_2B_, and A_3_, that are distributed throughout the body. Adenosine A_2A_R is expressed in ocular tissues of vertebrates [22-25]. Based on previous functional studies, we hypothesized that *A_2A_R* plays an important role in postnatal refractive development and thus is also a candidate susceptibility gene for high myopia.

By using single nucleotide polymorphisms (SNPs) as markers, association analysis is a powerful tool to find disease-related genes. Recently, various susceptibility genes have been identified by myopia- or high-myopia-association studies, including transforming growth factor-1 [26], catenin delta 2 [27], BH3-like motif containing cell death inducer [28], gap junction protein delta 2, actin alpha cardiac muscle 1 [29], and Ras protein-specific guanine nucleotide-releasing factor 1 [30]. However, most association analyses at either candidate gene or whole genome level have focused on tag SNPs; few functional SNPs (coding SNPs) have been tested. The purpose of this study was to determine if there are any significant associations between high myopia and SNPs in the *A_2A_R* exons in a Chinese population.

## Methods

### Subjects

We matched 175 unrelated subjects with high myopia (spherical power of less than −8.00 diopters [D]) in both eyes with 101 ethnically and socially similar, unrelated control subjects with refractive errors within ±1.00 D in each eye. All the subjects were from the Optometry Eye Hospital of Wenzhou Medical College, Wenzhou, China. Each of the participating subjects received a complete ocular examination including visual acuity (Topcon RM-8800; Topcon Corp., Tokyo, Japan), slit-lamp evaluation of the anterior segment (Topcon SL-1E Slit Lamp; Topcon Corp.), dilated fundus examination (Heine Omega 180 Binocular Indirect Ophthalmoscope; Heine Optotechnik, Herrsching, Germany) and axial-length measurements (Zeiss IOL Master; Carl Zeiss Meditec, Jena, Germany) [31]. Exclusion criteria included subjects with any known ocular, genetic, or systemic connective tissue disorders associated with myopia. Written consent was obtained from every subject after being fully informed of the purpose and procedures of the study. This study adhered to the tenets of the Declaration of Helsinki with subsequent revisions and was approved by the Human Subjects Ethics Committees of Wenzhou Medical College and the Eye Hospital, Wenzhou, China.

### DNA extraction and amplification

Genomic DNA for polymerase chain reaction (PCR) was extracted from 2 to 5 ml of peripheral venous blood from all participants. DNA was purified from lymphocytes according to the manufacturer’s instructions using a kit (BBI, Toronto, ON, Canada) [31]. The accession number for the *A_2A_R* sequence used for the construction of our primers was NT_011520. Four pairs of primers (Table 1) were used to amplify exon regions of *A_2A_R*. PCR was performed in a 50-μl volume with 31.25 μl double distilled H_2_O, 5 μl 10× PCR buffer (100 mM Tris-HCl pH 8.8, 500 mM KCl, 15 mM MgCl_2_, 0.8% Nonidet P40), 3.5 μl 25 mmol/l MgCl_2_, 4 μl 2.5 mmol/l deoxy-ribonucleoside triphosphate, 2 μl template DNA, 2 μl sense primer (all these ingredients were from Invitrogen, Carlsbad, CA), 2 μl antisense primer (Invitrogen, Shanghai, China), and 0.25 μl Ex Taq polymerase (Takara BIO Inc., Tokyo, Japan). The PCR reaction was initiated at 94 °C for 2 min, followed by 35 cycles (94 °C for 30 s, 65 °C for 30 s, 72 °C for 45 s), and ended at 72 °C for 10 min. PCR amplification products were purified using the PCR Cleanup Kit (Axygen Biosciences, Union City, CA).

**Table 1:** Primers used for the amplification and sequencing of *A_2A_R*.

**Fragment**	**Direction**	**Primer sequence**	**PCR product (bp)**	**Exon**
A_2A_1	S	5′- CGCCCTCTGCAGATGGTTCAGCT-3′	428	1
	AS	5′- GTTGCTGTTGAGCCACACGGCC −3′		
A_2A_2	S	5′- CCGTGCTGAGCCTGCCTGTCGTC −3′	434	2
	AS	5′- CCTGGCTCCGGGCACAGACCAA −3′		
A_2A_3	S	5′- CCCTGGCTTCTCAGATCTCTGAT −3′	577	2
	AS	5′- GGCGTAGATGAAGGGATTCACA −3′		
A_2A_4	S	5′- CTCATGTACCTGGCCATCGTCCTC −3′	611	2
	AS	5′- CTTCTTGCTGGGCCTCATGCTG −3′		

S, sense strand; AS, antisense strand.

### Single nucleotide polymorphism genotyping by sequencing

After the PCR amplification products were purified, nucleotide sequence analysis was performed with an ABI 3700 sequencer (Applied Biosystems Inc., Foster City, CA). Using Clustal W (version 3.0; European Bioinformatics Institute, Hinxton, Cambridgeshire, Great Britain), we compared all sequencing results to identify variations and to check the sequence map of mutable points to confirm the genotype. Positive results were further confirmed by reverse sequencing.

### Analysis of common single nucleotide polymorphisms

Allelic frequencies of all detected common SNPs were assessed for Hardy–Weinberg equilibrium (HWE). The frequency (P) of allele A was calculated as

$${\text{P}}_{\text{A}}=\left(\text{2}\times {\text{N}}_{\text{AA}}{\text{N}}_{\text{AB}}\right)/\left(\text{2}\times \text{N}\right)$$,

where N_AA_ is the number of wild types, N_AB_ is the number of heterozygotes, and N is the sample size of the group. The frequency of allele B was calculated as

$${\text{P}}_{\text{B}}=\text{1}–{\text{P}}_{\text{A}}$$.

The theoretical value of the three genotypes was calculated according to the following allele frequencies:

$${\text{N}}_{\text{AA}}\left(\text{E}\right)=\text{N}\times \left({{\text{P}}_{\text{A}}}^{\text{2}}\right)$$,

$${\text{N}}_{\text{AB}}\left(\text{E}\right)=\text{2N}\times {\text{P}}_{\text{A}}\times {\text{P}}_{\text{B}}$$,

$${\text{N}}_{\text{BB}}\left(\text{E}\right)=\text{N}\times \left({{\text{P}}_{\text{B}}}^{\text{2}}\right)$$,

where (E) is the expected value and N_BB_ is the number of homozygous mutations. The HWE for the genotype distributions was examined by the χ^2^ test in each group, where

$${\text{χ}}^{\text{2}}=\;{\left[{\text{N}}_{\text{AA}}–{\text{N}}_{\text{AA}}\left(\text{E}\right)\right]}^{\text{2}}/{\text{N}}_{\text{AA}}\left(\text{E}\right)+{\left[{\text{N}}_{\text{AB}}–{\text{N}}_{\text{AB}}\left(\text{E}\right)\right]}^{\text{2}}/{\text{N}}_{\text{AB}}\left(\text{E}\right)+{\left[{\text{N}}_{\text{BB}}–{\text{N}}_{\text{BB}}\left(\text{E}\right)\right]}^{\text{2}}/{\text{N}}_{\text{BB}}\left(\text{E}\right)$$.

The level of HWE was set at p>0.05. All samples were assumed to be from the same Mendelian population. Differences in the observed genotype, allelic frequencies, and heterozygous frequency (AB versus AA+BB) between the subjects with high myopia and the control subjects were also examined by the χ^2^ test. Statistical analyses were performed with the Statistical Package for the Social Sciences software (SPSS, version 13.0 for Windows; SPSS Science Inc., Chicago, IL). The power analysis for the χ^2^ test was run using Statistical Analysis Software (SAS, version 9.0; SAS Institute, Cary, NC).

## Results

For the high myopia group, the spherical power (OD; OS, mean±SD, D) was -15.44±6.06 D; -15.18±6.28 D; the astigmatism (OD; OS, mean±SD, D) was -1.60±1.21 D; -1.85±1.26 D; the axial length (OD; OS, mean±SD, mm) was 29.58±2.64 mm; 29.52±2.86 mm [31].

### Common and rare single nucleotide polymorphisms in *A_2A_R* exons

**Figure 1:**
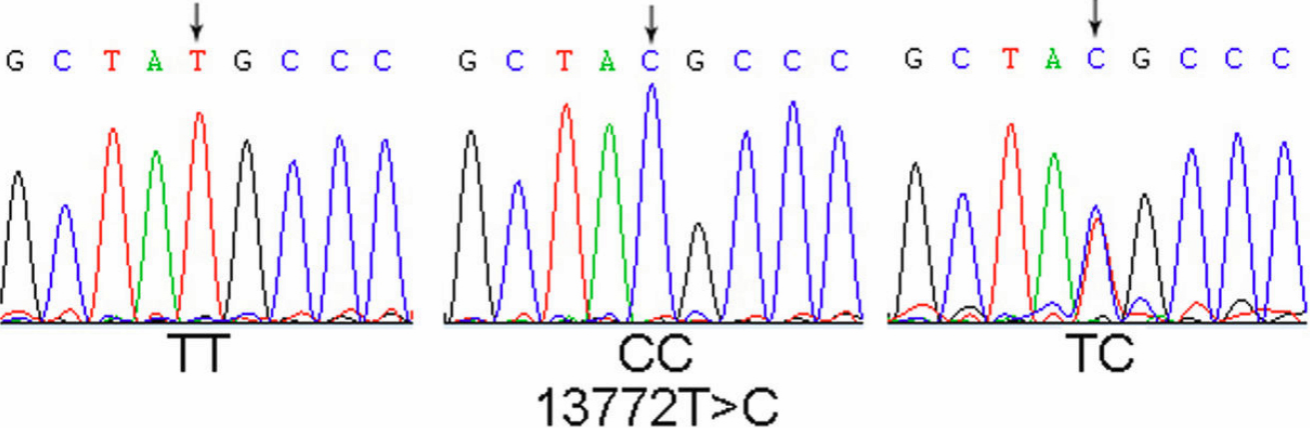
The common SNP 13772 T>C (rs5751876) was identified in *A_2A_R*. Although rs5751876, which codes for tyrosine, was located in coding regions, it did not affect the amino acid sequence.

**Figure 2:**
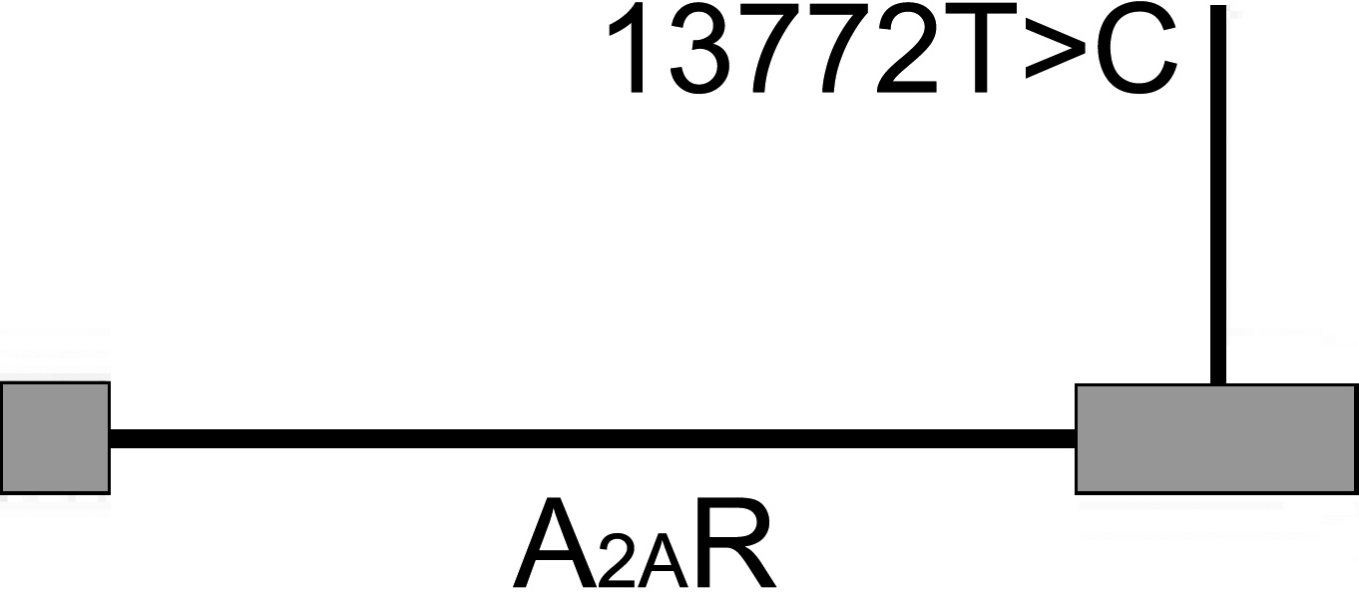
Schematic diagram of the single nucleotide polymorphism rs5751876 within *A_2A_R*. The gray rectangles represent exons, and the lines represent introns.

Purified PCR products specific to *A_2A_R* exons were sequenced to detect functional SNPs correlating to high myopia (Table 2). In total, six exonic substitutions were found (Table 3). Five of these, 5675 A>G, 5765 C>T, 13325 G>A, 13448 C>T, and 14000 T>A, were seen only in the control panel and had minor allele frequencies lower than 1%; as a consequence, no further analysis was conducted for these rare variants. In contrast, SNP 13772 T>C was common, with a frequency of 53.3% in all subjects (Figure 1, Table 3). Listed as rs5751876 (National Center for Biotechnology Information, Entrez SNP database, dbSNP Build 129), this is a synonymous substitution in codon Y361, which is located in the second exon of *A_2A_R* (Figure 2). The rs5751876 was also reported to be associated with anxiety in various investigations [32-34].

**Table 2:** Quantities of effective sequence results.

**Fragment**	**High myopia group (n)**	**Control group (n)**	**Total (n)**
A_2A_1	170	98	268
A_2A_2	163	101	264
A_2A_3	158	97	255
A_2A_4	161	96	257

**Table 3:** Observed frequency of the variations in high myopia and control groups.

**Variations**	**Polymorphism**	**High myopia group**	**Control group**	**Total**
5675	A→G	0/196 (0.00%)	1/340 (0.30%)	1/536 (0.19%)
5765	C→T	0/196 (0.00%)	2/340 (0.59%)	2/536 (0.37%)
13325	G→A	0/194 (0.00%)	1/316 (0.32%)	1/510 (0.20%)
13448	C→T	0/194 (0.00%)	1/316 (0.32%)	1/510 (0.20%)
14000	T→A	0/192 (0.00%)	2/322 (0.62%)	2/514 (0.39%)
13772 (rs5751876)	T→C	104/192(54.17%)	170/322 (52.80%)	274/514 (53.31%)

### Lower heterozygous frequency of rs5751876 in subjects with high myopia

To examine the association between rs5751876 and high myopia, we compared the variation frequency of this SNP in subjects with high myopia and control subjects by the χ^2^ test. No significant difference was observed between the case and control groups by either genotype frequency test (p=0.11) or allele frequency test (p=0.76).

However, the genotype distribution of rs5751876 in subjects with high myopia deviated significantly from HWE (p=0.0016), while no such deviation was seen in control samples. Further testing on the distribution of homozygotes and heterozygotes demonstrated a significantly lower frequency of the heterozygous genotype in subjects with high myopia compared to that of the control subjects (p=0.033, Table 4). This result suggests a possible association of *A_2A_R* with high myopia in Chinese population.

**Table 4:** Genotype frequency of 13772 T>C (rs5751876).

Genotype*	**High myopia group**	**Control group**	**Total**
TT	26/96 (27.08%)	35/161 (21.74%)	61/257 (23.74%)
TC	36/96 (37.50%)	82/161 (50.93%)	118/257 (45.91%)
CC	34/96 (35.42%)	44/161 (27.33%)	78/257 (30.35%)

TT, genotype with homozygous normal allele; TC, genotype with heterozygous sequence alterations; CC, genotype with homozygous sequence alterations. *Based on association test using χ^2^ statistics, *P_overall_*=0.11 and *P*_TC versus TT+CC_=0.03.

## Discussion

Two facts prompted us to conduct this investigation to determine if coding SNPs in the *A_2A_R* gene contributes to the development of high myopia. First, previous association studies on high myopia mainly focused on tag SNPs [35]; few functional variants (e.g, coding SNPs) were identified for their association with high myopia [36]. Furthermore, myopic changes observed in knockout mice suggest that certain genes may cause or contribute to refraction error in humans. In particular, myopic changes occur in early growth response 1 knockout mice [37] and in *A_2A_R* knockout mice [20]. Although these pathologic abnormalities are similar to those in humans with high myopia [1-3], no mutation was found in early growth response 1 exons in Chinese subjects with high myopia [37], and correlation between *A_2A_R* and high myopia remains unknown.

In our sequencing results only one SNP (rs5751876) shows a statistical power for association analysis (minor allele frequency=46.7%). Previous association studies have linked this locus to susceptibility of anxiety [32-34]. Despite no observed correlation in the genotype and allele frequency test, the significant deviation from HWE and the lower heterozygote genotype frequency in case but not in control subjects suggest an association between this SNP and high myopia, at least in Chinese population. Moreover, the C allele frequency of rs5751876 in our individuals (53.3%) was lower than that in Europeans and Africans (72.2% and 60%, respectively, dbSNP), further implying different signals of *A_2A_R* among populations. Expanding the sample size may provide stronger signals for the association of this SNP to high myopia.

Being a synonymous substitution, the association signal of rs5751876 to high myopia could be explained in two ways. First, this substitution may change the codon of Y361 (TAC to TAT), which in turn affects the translation efficiency by tRNA-dependent codon usage bias [38]. Another possibility may be a high linkage disequilibrium of this SNP with rs2298383 (D’=1) in an upstream transcription factor binding site [33]. Therefore, rs5751876 is perhaps a marker for surrounding SNPs corresponding to *A_2A_R* expression.

Adenosine A_2A_R is expressed in ocular tissues and participates in eye growth. As described above the relevance of *A_2A_R* to high myopia is supported by a line of evidence from cellular and animal studies [20]. These findings as well as our genetic results indicate that adenosine and adenosine receptors contribute to the development of high myopia. The mechanism(s) by which *A_2A_R* and/or other adenosine receptors contribute to myopia is currently unknown. Functional analysis will help elucidate the molecular pathways of *A_2A_R* and determine if pharmaceutical intervention targeting adenosine receptors may inhibit development of myopia.
